# Physicochemical and biological analysis of river Yamuna at Palla station from 2009 to 2019

**DOI:** 10.1038/s41598-022-06900-6

**Published:** 2022-02-21

**Authors:** Pankaj Joshi, Akshansha Chauhan, Piyush Dua, Sudheer Malik, Yuei-An Liou

**Affiliations:** 1 Department of Civil Engineering, SRM IST, Delhi-NCR Campus, Ghaziabad, India; 2 Centre for Space and Remote Sensing Research, National Central University, Taoyuan City, Taiwan; 3 Department of Engineering, University of Technology and Applied Sciences, Suhar, 311 Sultanate of Oman; 4 Department of Chemistry, SRM IST, Delhi-NCR Campus, Ghaziabad, India; 5 National Museum Marine Science and Technology (NMMST), No. 367, Beining Rd., Zhongzheng Dist., Keelung City, 202010 Taiwan

**Keywords:** Environmental sciences, Hydrology

## Abstract

Yamuna is one of the main tributaries of the river Ganga and passes through Delhi, the national capital of India. In the last few years, it is considered one of the most polluted rivers of India. We carried out the analysis for the physiochemical and biological conditions of the river Yamuna based on measurements acquired at Palla station, Delhi during 2009–19. For our analysis, we considered various physicochemical and biological parameters (Dissolved Oxygen (DO) Saturation, Biological Oxygen Demand (BOD), Chemical Oxygen Demand (COD), Total Alkalinity, Total Dissolved Solids (TDS), and Total Coliform. The water stats of river Yamuna at Palla station were matched with Water Standards of India, United Nations Economic Commission for Europe (UNECE), and World Health Organization (WHO). Maximum changes are observed in DO saturation and total coliform, while BOD and COD values are also seen higher than the upper limits. Total alkalinity rarely meets the minimum standards. TDS is found to be satisfactory as per the standard limit. The river quality falls under Class D or E (IS2296), Class III or IV (UNECE), and fails to fulfill WHO standards for water. After spending more than 130 million USD for the establishment of a large number of effluent treatment plants, sewage treatment plants, and common effluent treatment plants, increasing discharges of untreated sewage, partially treated industrial effluents and reduced discharge of freshwater from Hathnikund are causing deterioration in water quality and no major improvements are seen in water quality of river Yamuna.

## Introduction

Water is the main need of human life. The majority of ancient civilizations were developed on the bank of major rivers across the world. Rivers fulfill the major demands of the freshwater supply from drinking to agriculture. In the northern parts of India, the Yamuna River basin is ranked the second largest basin after the Ganga River. It is the second-largest tributary of the river Ganga (the longest river of India) with a total catchment area of 345,848 km^2^ and it originates at Yamunotri Glacier, Uttrakhand, India at a height of 6387 m^1^. It covers a total distance of 1376 km through four major states of India: Uttarakhand, Haryana, Delhi, and Uttar Pradesh, and finally confluences with river Ganga at Triveni Sangam, Prayagraj, Uttar Pradesh. Although it does not flow through Himachal Pradesh but receives water via the river Tons (which originates in Himachal Pradesh). The other tributaries of the river Yamuna are Chambal, Sindh, Betwa, and Ken^2,3^. The water abstracted from the river is mostly used for irrigation (about 94%), while 4% for domestic water supply and the remaining 2% for industrial and other uses^4^.

**Figure 1:**
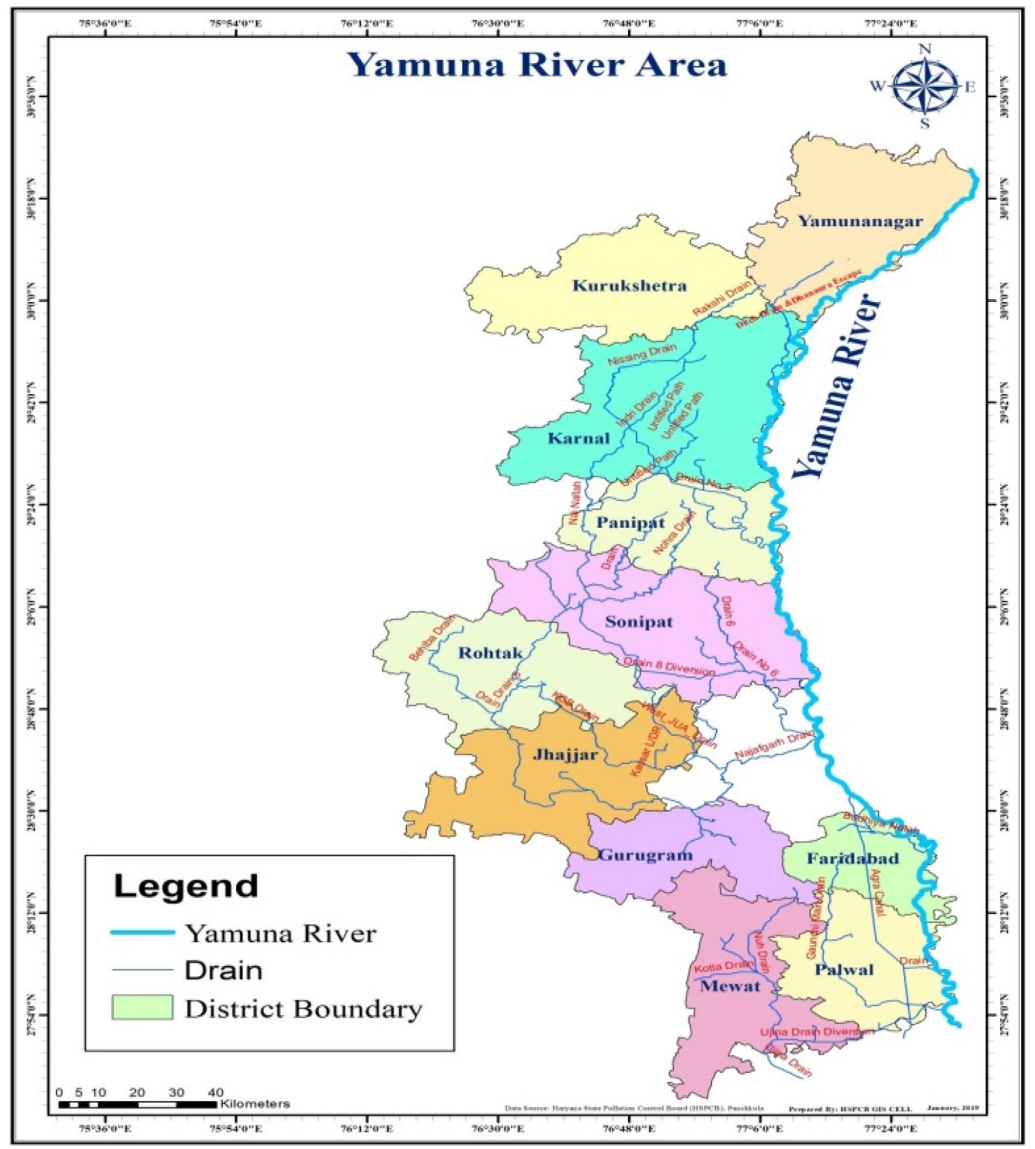
River Yamuna in Haryana. (*Source*: HSPCB).

**Figure 2:**
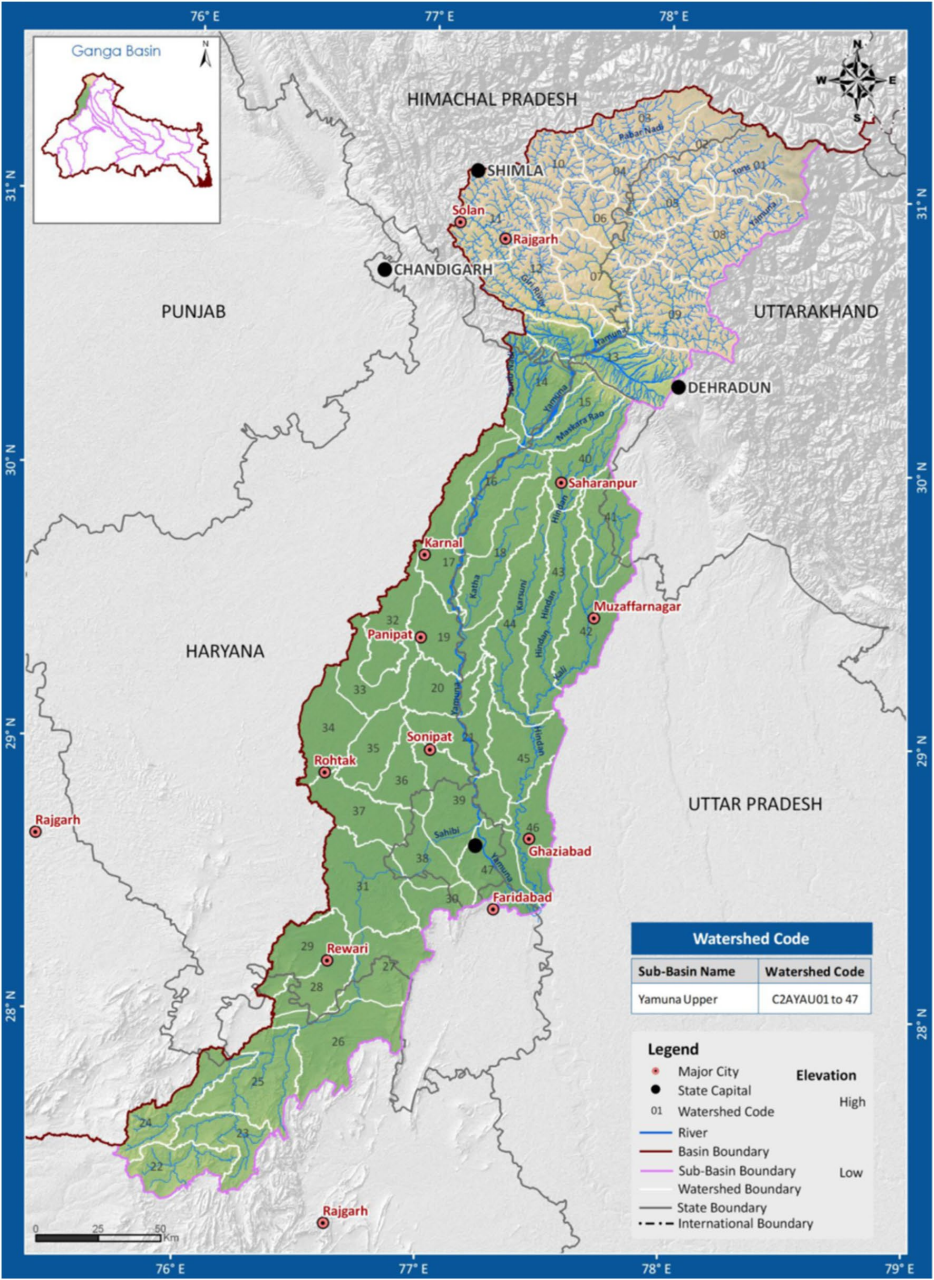
Upper river Yamuna basin area. (*Source*: WRIS).

During the last few decades, Yamuna has been considered one of the most polluted rivers of India. Discharge from industries, partially or untreated sewage, and agricultural waste are the main sources for the river Yamuna degradation^5–7^. Almost 85% of the total pollution in the river Yamuna is due to domestic sources mainly from urban cities Sonipat, Panipat, Delhi, Ghaziabad, Mathura, Agra, Etawah, and Prayagraj. Industrial zones at various places like Yamunanagar, Panipat, Ghaziabad, Delhi, Noida, Faridabad, and Baghpat which are in the upper Yamuna basin (Fig. 1), comprise industries like Oil refineries, distilleries, pulp, pharmaceutical, chemical, electroplating, weaving, and sugar, and contribute to the degradation of Yamuna water quality significantly^8,9^. According to Kumar et al.^10^, Delhi leads the list of cities with 79% pollution load in river Yamuna followed by Agra and Mathura with a contribution of 9% and 4%, respectively, whereas a pollution load of 2% by Sonipat and Baghpat. The annual mixing of sewage from domestic and industrial sources in the Yamuna River basin is about 9.63 km^3,11^. In the last few decades, a sudden rise in the built-up and cropland areas is observed in the Yamuna River basin (Fig. 2). Kumar et al.^10^ suggested a rise of 100% in the urbanization in Haryana and Rajasthan states and significant fall is observed in wetland, grassland, water bodies and forest areas of Yamuna River basin. The green revolution in India helped rise in the productivity of various crops, but the major water supply to the crop depends on the groundwater. The DO level of water in Delhi stretch shows a sudden fall due to high carbon level so that most of the time the river can not sustain fishery.

Parween et al.^12^ showed the positive rise in the potassium and nitrate that affected the Yamuna River basin. Domestic waste consists of mainly organic matter and micro-organisms along with detergents, grease and total salts mixed in river Yamuna through various drainages in National Capital region. Lokhande and Tare^3^ have shown rise in the flow rate of Yamuna during non-monsoon months due to the sewage water. Industrial effluents are the main source of heavy metal pollution like Cd, As, Cr, Fe and Zn with other inorganic and organic wastes adding to pollutant inventory^10^. According to National Capital Region Planning Board (NCRPB) report, sewage generation in Haryana was 374 MLD in 2001, and 599 MLD in 2011, whereas the sewage treatment capacity was 164 MLD in 2001 and 199 MLD in 2011. State monitoring committee appointed by National Green Tribunal (NGT) 2019 suggested that Haryana discharged 1140 MLD of untreated or partially treated sewage per day into river Yamuna, also 1268 industrial units discharged 138.75 MLD partially treated and another 827 units discharged 48.319 MLD of treated effluents per day in Yamuna River^13^.

Central Pollution Control Board (CPCB) is responsible for controlling the various sources of pollution in India and also monitoring water quality of the rivers with the State Pollution Control Board (SPCB)^4,5,14,15^. CPCB started national water quality monitoring in 1978 under Global Environmental Monitoring System (GEMS), followed by the Monitoring of Indian National Aquatic Resources (MINARS) in 1984, and helped reduce river pollution via National Water Quality Monitoring Programme (NWMP)^16^. Central Water Commission (CWE) is monitoring the water quality of all the major river basins in India through 519 water quality sites and 33 water sampling stations. According to CWC, the water quality of river Yamuna is monitored at 18 different stations of which 12 are manual and 6 are telemetry stations, starting from Naugaon (N-30.78, E-78.13) at Uttarakhand as the first river point station to the last river point station Pratappur (N-25.37, E-81.67) Uttar Pradesh.

Due to degradation in the water quality of the Yamuna River, Yamuna Action Plan (YAP-I) was launched in 1993 by the Ministry of Environment and Forests (MoEF), India to rejuvenate the Yamuna River especially in the Delhi segment having maximum pollution load. Haryana and Uttar Pradesh were also included along with Delhi in YAP-II in 2003. YAP-III, with an estimated cost of Rs.1656 crore, was launched in 2018 as an integrated component of the Namami Gange Mission^17^. The river Yamuna is analyzed monthly, seasonally, and yearly for its physical, chemical, and biological properties in previous research for various states and cities influenced by it or small stretches of river^18–21^. A sufficient amount of literature is available for the water quality of Yamuna and most of the analysis focused on the Delhi and stations lie after the Delhi’s. Kumar et al.^10^ discussed the variability of various water quality parameters from 1999 to 2005 to investigate the relationship between environmental parameters and pollution sources. Kaur et al.^22^ discussed the impact of industrial development and land use/land cover changes over the Yamuna water quality in Panipat, which is located between the Hathnikund and Baghpat stretch. The sampling was carried out during February, July, October, and December, 2018 to observe the variations in the quality of water and the impact of pollutants on the river Yamuna. Patel et al.^23^ carried out the water quality analysis over the Yamuna River using satellite and remote sensing during the lockdown period and compared it with the water quality parameter before the lockdown. The analysis was carried out from January 2020 to April 2021. The impact of lockdown on the water quality was discussed assuming that the industrial waste and the other pollutants sources were reduced during the time of lockdown. Paliwal et al.^24^ carried out the modeling analysis of the Yamuna water quality for the Delhi stretch. They emphasize the inflow of untreated water and effluents from various drains in the Yamuna in the Delhi stretch using QUAL2E-UNCAS before 2007. They also highlighted the need for common treatment plants and a rise in freshwater supply. Krishan et al.^25^ conducted the groundwater study near the bank of the Yamuna in the Agra and Mathura districts. The study area is located downstream of Delhi and affected by severe falls in water quality. The change in the water quality at Agra and Mathura was discussed and the assessment of the treated water after the filtration was discussed. Kaur et al.^26^ investigated the Yamuna water quality at the Delhi stretch and one site located near the Yamunotri. The analyses were implemented during March and October for 2017 and 2018. They have discussed the impact of the inflow of pollutants from the Delhi and NCR regions in the Yamuna. Jaiswal et al.^21^ carried out the multivariate study of the Yamuna river water to study the river water quality across the whole stretch. The samples were collected from July to October and November to June for 2013 and 2014. The analysis suggested that the water quality of Palla was suitable for drinking during the study period. We found that these analyses were mostly carried out for a short period in recent years. Some long-term analyses were carried out before 2007. So, there is a dire need for long-term analysis in recent years. In recent times, Lokhande and Tare^3^ performed the first long-term analysis of various water quality parameters of the river Yamuna and discussed the trends of various parameters. Due to classified data, Lokhande and Tare^3^ were not able to quantify the monthly variations of various water parameters. Hence, these analyses, lack the long-term variability discussion and quantitative changes. In the current study, we conducted monthly and annual mean analysis of various physical and biological parameters, including Biological Oxygen Demand (BOD), Chemical Oxygen demand (COD), Dissolved Oxygen (DO) Saturation, Total Alkalinity, Total Dissolved Solids (TDS), Total Coliform and average rainfall from 2009 to 2019 at Palla station located at northwestern Delhi. We have shown the quantitative change in the physiochemical and biological parameters of the river Yamuna at the Palla station, which is mostly affected by the pollutants load of the watershed of Haryana. We compared the results with the water quality guidelines of national and international standards given in Table 1 to figure out the changes in water quality of river Yamuna at Palla station in the last 11 years. The current water quality at Palla is not suitable for drinking and sometimes not good for agricultural purposes due to the high influx of water pollutants.

**Table 1:** Water quality standards by BIS, UNECE and WHO.

Parameters	Class										
	IS 2296:1992					UNECE 1994^27^					WHO
	A	B	C	D	E	I	II	III	IV	V	
DO (%) un-stratified water	–	–	–	–	–	90–70	70–50	50–30	30–10	< 10	–
DO (mg/l)	≥ 6	≥ 5	≥ 4	≥ 4	–	> 7	7–6	6–4	4–3	< 3	5
BOD (mg/l)	≤ 2	≤ 3	≤ 4	–	–	–	–	–	–	–	–
COD (mg/l)	–	–	–	–	–	< 3	3–10	10–20	20–30	> 30	10
TDS (mg/l)	≤ 500	–	≤ 1500	–	≤ 2100	–	–	–	–	–	500
Total alkalinity (mg/l)	200	–	–	–	–	> 200	200–100	100–20	20–10	< 10	200
Total coliform (MPN/100 ml) max	50	500	5000	–	–	–	–	–	–	–	0

## Study area

**Figure 3:**
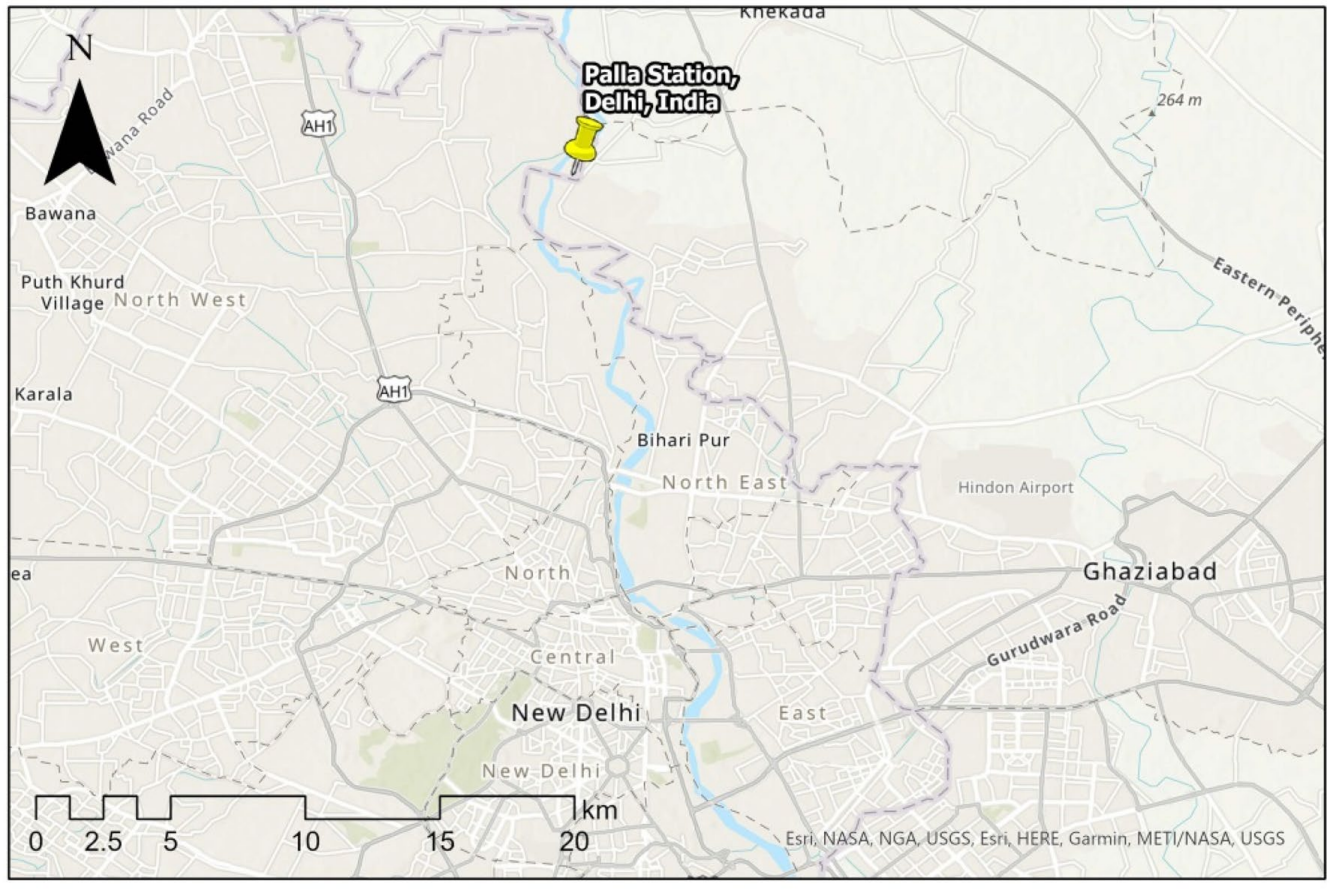
Location of Palla station. The base image is provided by ESRI and projection is done using ArcGIS Pro.

The river Yamuna enters National Capital Territory (NCT) at approximately 1.5 km before village Palla, which is 23 km upstream of the Wazirabad barrage. Palla station (N-28.82 and E-77.22) is a manual type station with zero gauges at 206 m. Before entering NCT at Palla, the river Yamuna traveled about 393 km from its source and about 220 km from the Hatnikund barrage. According to Haryana State Pollution Control Board (HSPCB), the numbers of industries in Yamunanagar, Kernal, Panipat, and Sonipat are 142, 9, 346, and 503, generating effluent of 16,420.90, 26.00, 65,696.97, and 15,668.50 KLD, respectively, up to August 2019. As in Fig. 3, several drains of Haryana state including 3 major drains at Dhanaura escape, Main Drain No.2, and Drain No. 8 also fallout in river Yamuna before reaching Palla^28^ (Figs. 3, 11).

### Data retrieval

Water quality data of Yamuna River at Palla station was obtained from Water Resources and Information System (WRIS), India, which is a centralized platform, acting as a database related to all water resources at the national or state level. It was initiated by CWC along with the Ministry of Water Resources, Ministry of Jal Shakti, and Indian Space Research Organization (ISRO) in 2008 to provide a single-window solution to all water resources data and information in a standardized national GIS framework^29^. Depending upon the availability of monthly average data, the parameters BOD, COD, DO saturation, total alkalinity, TDS, and total coliform at Palla station were analyzed during the period from January 2009 to December 2019. The rainfall data was procured from the Modern-Era Retrospective analysis for Research and Applications, Version 2 (MERRA-2) and available on the website http://www.soda-pro.com/web-services/meteo-data/merra. The spatial resolution of data is 0.50° × 0.625° and the time step ranges from 1 min to 1 month^30,31^. We also included the water discharge data in the current study of three locations Hathnikund Barrage, Baghpat, and Delhi Railway Bridge (DRB) stations. The freshwater in river Yamuna reached to the Palla station is controlled at the Hathnikund Barrage. Baghpat station is located just before the Palla station (no data is available at Palla station) and DRB is located after the Palla station. These stations are chosen based on the availability of data and the locations to show the water discharge in the Yamuna. The water discharge data of this location is taken from the Central Pollution Control Board of India (https://yamuna-revival.nic.in/wp-content/uploads/2020/07/Final-Report-of-YMC-29.06.2020.pdf).

### Ethics approval and consent to participate

The paper did not involve any human participants.

## Result and discussion

Analysis of various parameters related to the water quality of river Yamuna at Palla station was carried out.

### Rainfall

**Figure 4:**
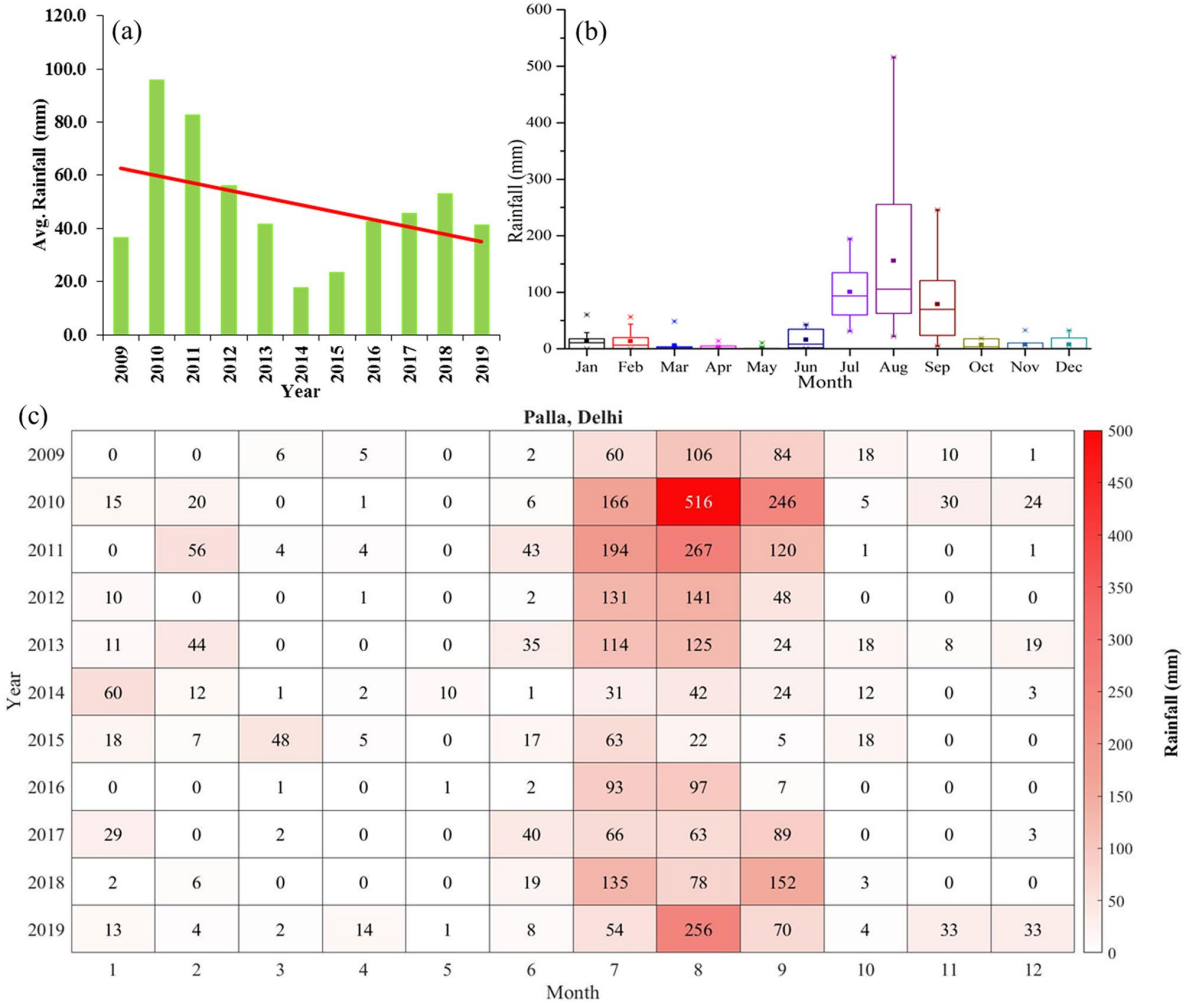
(**a**) Yearly mean rainfall at Palla station and adjacent region from 2009 to 2019. The red line shows a linear trend of rainfall. (**b**) Rainfall monthly distribution at Palla station from 2009 to 2019. (**c**) Monthly mean variation of rainfall from 2009 to 2019.

The annual mean variation in rainfall at the Palla region (Fig. 4a) suggested that the highest annual mean rainfall (95.8 mm) occurred during the year 2010. After 2010, the yearly average rainfall continuously declined every year till 2014 with a mean value of 17.9 mm. Although from 2014 to 2018, a rise in average rainfall was observed each year and during 2018, the average rainfall was calculated as 53.1 mm, but the linear trend illustrated a sharp decline in average rainfall between 2009 and 2019 as shown in Fig. 4a.

The box plot in Fig. 4b shows the rainfall data of each month during 2009–2019. The distribution suggested that the monthly mean rainfall variations were highest during August along with July and September. May was an almost driest month along with April and March as the rainfall was minimum during these months. The highest values of average rainfall for August, September, and July were 515.28, 233.45, 207.99 mm respectively, whereas these months’ minimum average rainfalls were 101.74, 61.95, and 72.76 mm, respectively, during 2009 and 2019. The median values of rainfall during March, April, October, and November were below 10 mm and, during January, February, June, and December, the median values were between 10 and 20 mm, while the median values for July, August, and September were above 60 mm.

The monthly mean variation of rainfall (2009–19) further elaborates the stats of rainfall in the Palla region. From 2009 to 2019 rainfall data showed various ups and downs in monthly rainfall (Fig. 4c). In India, the onset of monsoon is observed during the start of June each year in the coastal part of India, which lasts approximately for a period of four months (June to September) each year. The northern parts of India receive major rainfall during this period, which is known as the monsoon season. During June, the average rainfall remained lower than the decadal mean during 2014–2019 except for 2017. During July, we observed the same deficit of rainfall during 2014–2019 except in 2018. The major deficit in rainfall was observed for August from 2012 to 2018 whereas, during August 2019, the rainfall remained higher than the decadal mean value. During September, the rainfall deficit was observed from 2012 to 2016. Hence, during 2012–2017, we observed a significant fall in the monsoon rainfall. The winter rainfall is also observed during January and February in northern parts of India. We also observed a significant fall in the winter rainfall for January and February since 2014. Yamuna River before Palla station receives runoff water from cities like Sonipat, Panipat, Karnal, and Yamunanagar. The rainfall statistics of Haryana analyzed by India Meteorological Department (IMD) between 1989 and 2018 showed annual rainfalls of 1053.5, 578.8, 573.9, and 495.3 mm at Yamunanagar, Sonipat, Karnal, and Panipat districts, respectively^32^. During the monsoon season, sudden changes in physicochemical and biological parameters were observed by various researchers. The addition of monsoon runoff in rivers dilutes the industrial effluent and sewage, causing the decline in parameters like BOD, COD, alkalinity, pH, and conductivity^33–36^. The catchment area of Yamuna is the smallest in Delhi. The size of the catchment area of the river is an important factor for the dilution of anthropogenic waste in river water. The small catchment area increases soil components in the river and makes difficult the dilution process of anthropogenic waste^37^. These conditions can further affect the river water quality. Hence, we further analyzed the variations in the various physical and biological parameters for each month from 2009 to 2019.

### Dissolved oxygen (DO) saturation

Dissolved Oxygen (DO) saturation is a vibrant parameter for aquatic system health determination. The pollutants like sewage, soil, agricultural runoff, and other organic pollutants can reduce the DO saturation of water^38^ and low DO saturation can impact the life of major aquatic organisms. The water having DO < 5% of saturation lies in an extremely severe pollution region; DO between 5 and 10% of saturation lies in severe pollution; DO in a range of 10–70% represents moderate pollution, whereas DO above 70% indicates slightly or no pollution condition. Heavy pollution load due to untreated sewage and industrial effluents are the main causes of decreasing DO concentration^39^. Value of DO saturation is also affected by the change in water salinity (chlorine), temperature, and air pressure.

**Figure 5:**
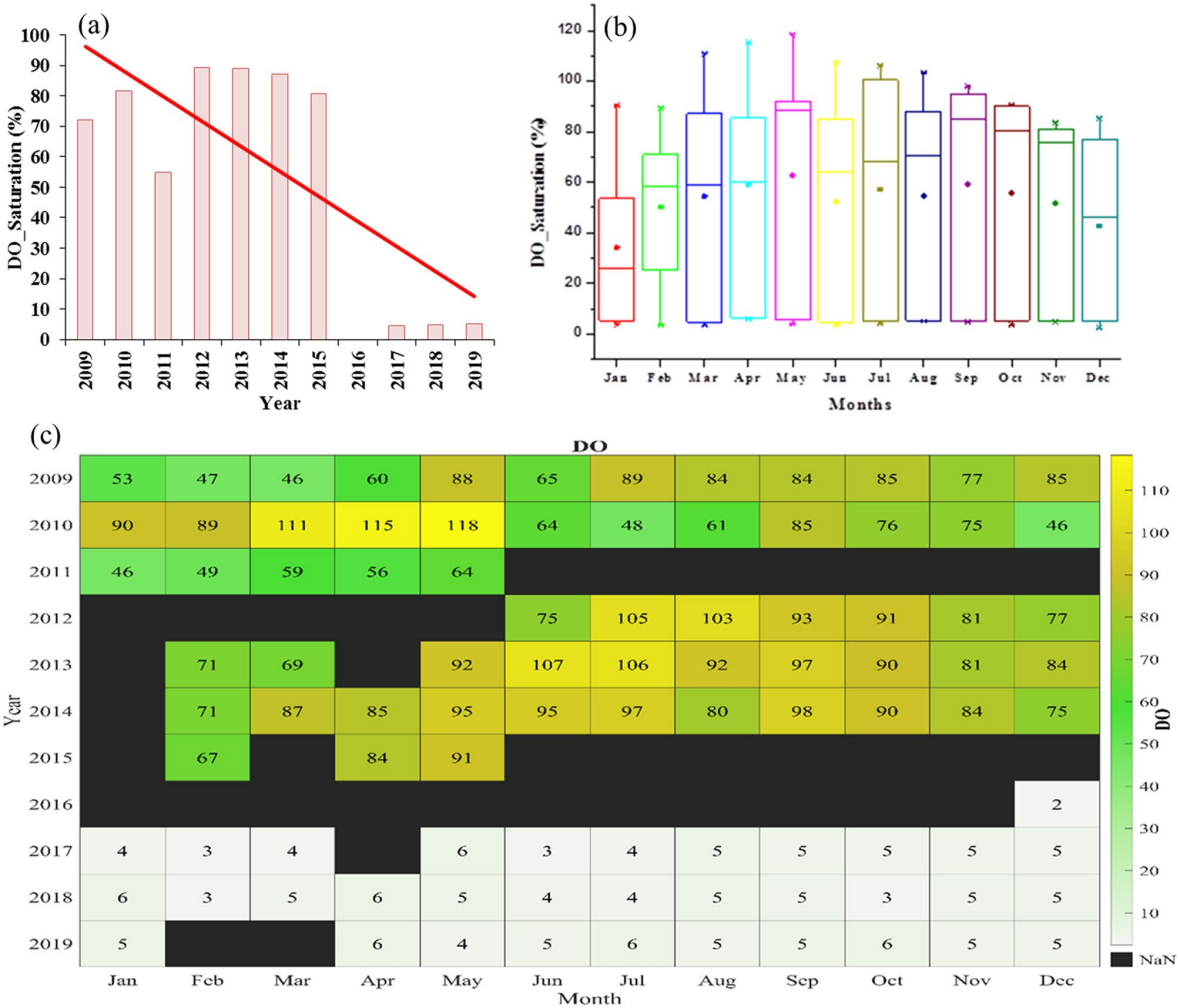
(**a**) Yearly variation of DO saturation from 2009 to 2019. The red line shows a linear trend of DO saturation. (**b**) Monthly distribution of DO saturation at Palla station from 2009 to 2019. (**c**) Monthly mean variation of DO (%) saturation from 2009 to 2019.

Increasing pollution in the Yamuna River caused a decrease in DO saturation concentration along with an increase in temperature and salinity of water^3^. Figure 5 shows the yearly variation of DO saturation, and monthly distribution and mean variation of DO saturation at Palla station from 2009 to 2019. In recent years, the annual mean value of DO saturation during 2017–19 was found to be 4.81%. From 2009 to 2011, the mean DO saturation was found to be 69.49% with a maximum value (81.58%) in 2010. From 2012 to 2015, the yearly mean DO saturation was 86.48%. During this period, a small decrease was seen between 2013 and 2015. In recent years, the DO saturation reached critically low values to the average value before 2015 as demonstrated in Fig. 5a. These conditions indicate the rise in pollutants in the river Yamuna.

The monthly distribution of DO saturation during 2009–2019 is shown in Fig. 5b with boxes ranging from 25 to 75%. During May, the maximum median value was 88%, whereas in January minimum median value was about 25%. Similarly, the maximum monthly mean value was 62.7% during May, while the minimum mean value was 34.1% during January. Similarly, maximum DO saturation of 118% was perceived for May, while a minimum of 2.4% was observed for December. The monthly mean distribution suggested that the mean DO saturation plunged between 50 and 60% with a slight growth in trend from January to December. We further showed the monthly mean values of DO saturation in Fig. 5c. From 2009 to 2015, the values suggested no major changes with monthly values well above 50, but just after 2017, a sudden fall was observed in the DO saturation each month. We observed 10 times fall in DO saturation just after 2017. During 2009–14, the DO saturation of Yamuna River at Palla station was Class I category of international standards for surface water of UNECE, but during 2016–19 its quality degraded to Class IV due to regular fall in the DO values of the river water. The lack of fresh water and rising carbon concentration have affected the DO concentration significantly.

### Biological oxygen demand (BOD)

**Figure 6:**
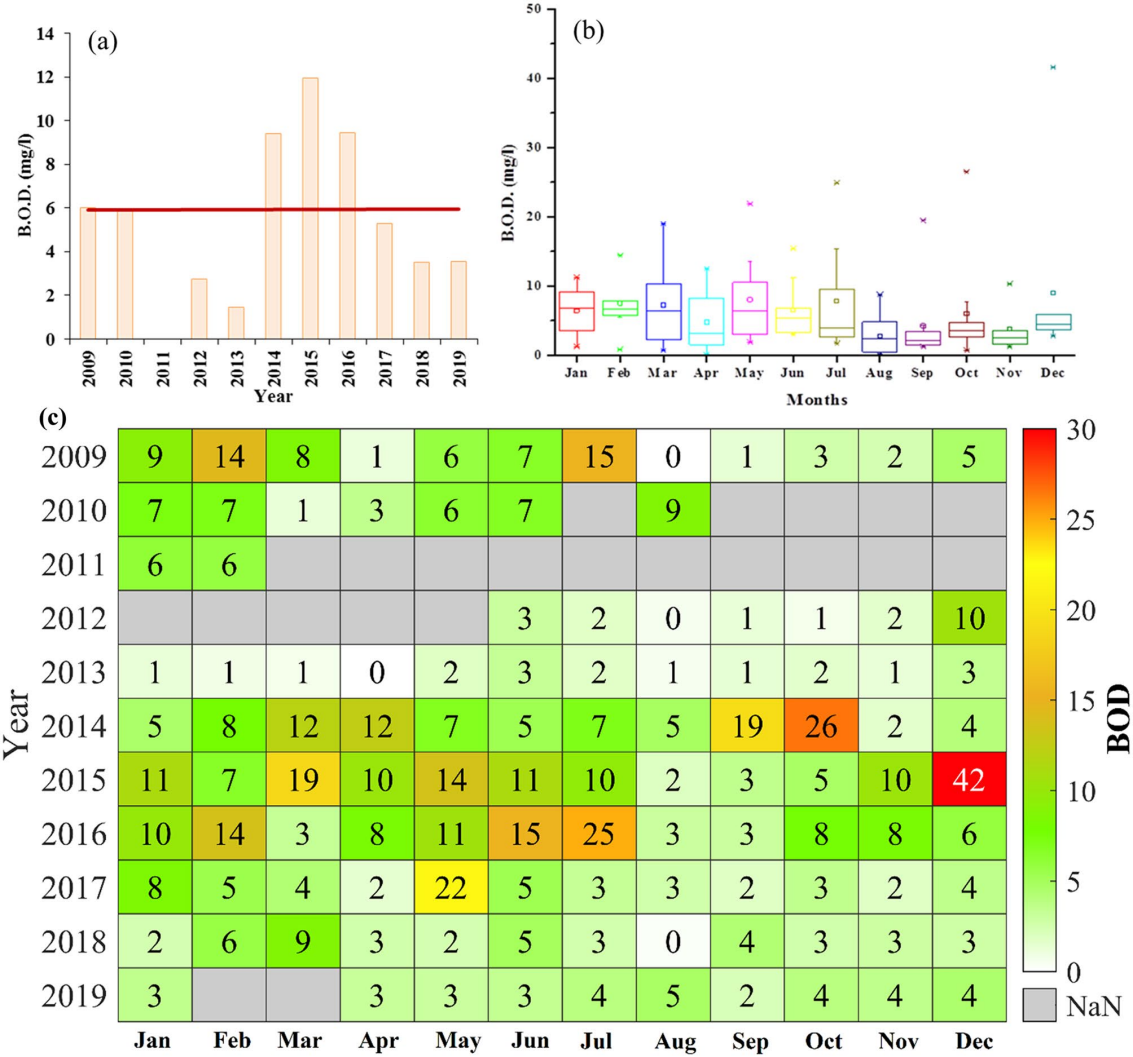
(**a**) Temporal variation of yearly mean BOD from 2009 to 2019. The red line shows a linear trend of BOD. (**b**) Monthly distribution of BOD at Palla station from 2009 to 2019. (**c**) Monthly mean variation of BOD from 2009 to 2019.

Biological Oxygen Demand (BOD) is one of the methods to assess the quality of water by calculating the oxygen requirement for decomposition of its organic matter. The yearly variations in BOD at Palla station during 2009–19 are shown in Fig. 6a. The yearly mean BODs in 2009 and 2010 were estimated to be around 6 mg/l, while, during 2013 and 2012, the least values of BOD (1.4 and 2.7 mg/l, respectively) were found. The year 2015 showed the highest yearly mean BOD value of around 12 mg/l followed by years 2014 and 2016 with a value of about 9.5 mg/l. The decline in yearly mean BOD was observed from 2015 to 2019. In particular, a BOD of 3.5 mg/l was perceived for years 2018 and 2019. The monthly variation of BOD during 2009–19 was shown in Fig. 6b, where the interquartile range for each month was between 25 and 75 percentiles. The first quartile for all months lay below the range between 0 and 5 mg/l, with outliers for months May, July, October, and December with BOD above 20 mg/l. January and February had the highest median value of BOD at 6.7 mg/l, while September had the least median value of 2.1 mg/l.

The median value for April, June, July, October, and December lay between 3 and 5 mg/l. The monthly mean BOD during 2009–19 represented that BOD was maximum in December (8.9 mg/l) followed by the second highest mean value of 8 mg/l in May. The minimum mean value of 2.7 mg/l was found in August, while in April, September, October, and November, the mean BOD lay between 3 and 6 mg/l. Mean BOD from January to March and in July was in the range of 6–7.5 mg/l.

The fluctuation in BOD was observed during 2009–19 in Fig. 6c. In January, February, and March, a decrease in BOD occurred during 2009–13, while in May, June, and July increase in BOD was seen during 2013–16. In August, September, and October, BOD mostly fell below 5 mg/l throughout the study period. The highest BOD values were measured in December 2015, October 2014, and July 2016 (41.6, 26.5, and 24.8 mg/l, respectively). The decreasing trend was observed for January, February, and July, with a rising trend for March, April, May, and November. From 2014 to 2016, the BOD values were found to be several times higher than the acceptable limits. In March and April, BOD values were less than 4, and else BOD was higher than 4 even in monsoon months. In years 2018–19, BOD is observed to be mostly ≤ 4 mg/l. However, the overall BOD values suggest that the water quality of Yamuna mostly lay beyond the C category (BIS) as the BOD values are mostly > 4 mg/l. To attain river quality standard it has to be ≤ 3 mg/l.

### Chemical oxygen demand (COD)

**Figure 7:**
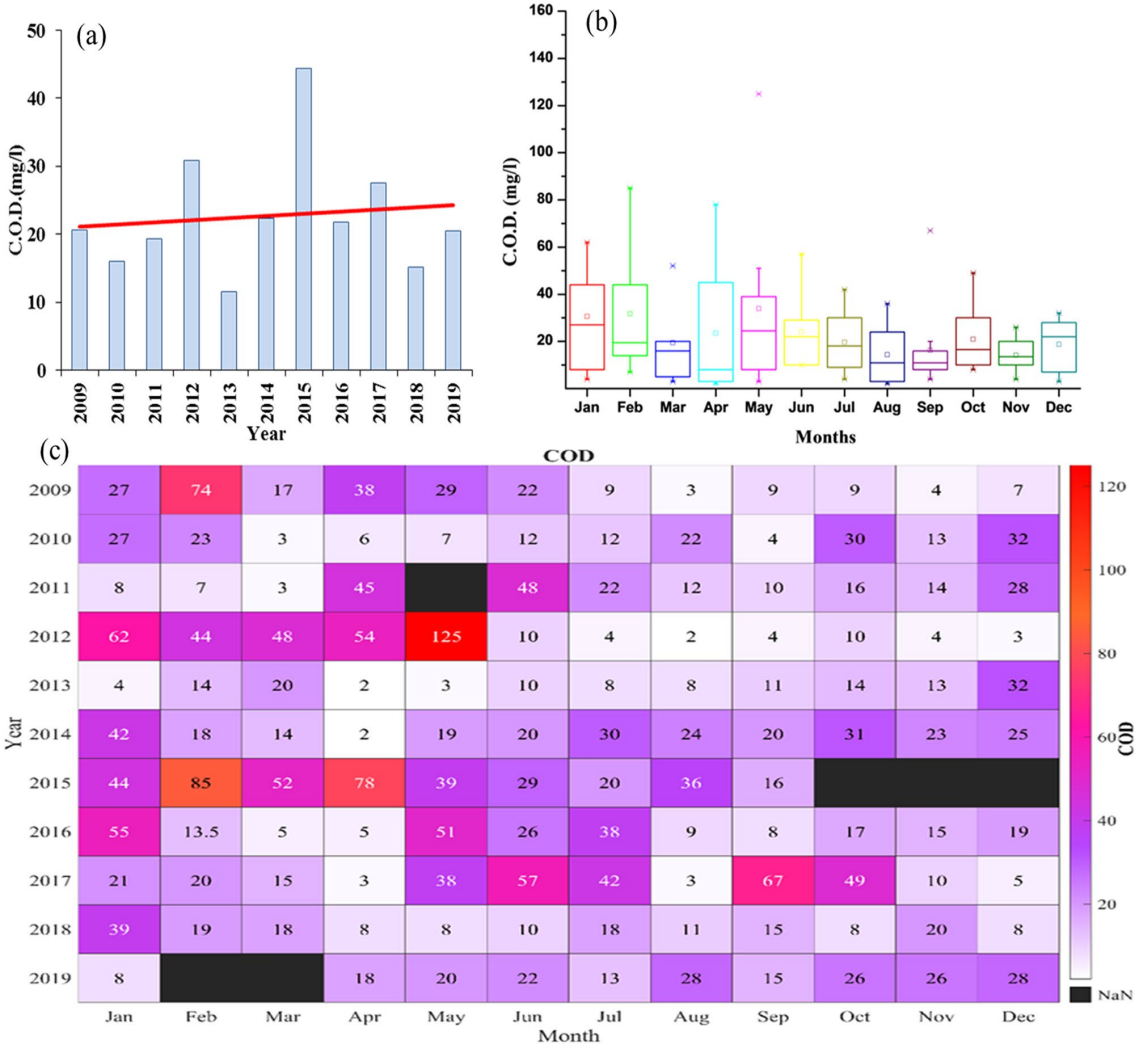
(**a**) Yearly variation of COD from 2009 to 2019. The red line shows a linear trend of COD. (**b**) Monthly distribution of COD saturation at Palla station from 2009 to 2019. (**c**) Monthly mean variation of COD from 2009 to 2019.

Chemical Oxygen Demand (COD) determines the amount of oxygen required for the oxidation of organic matter present in water. We have shown the changes in COD at Palla station in Fig. 7. Yearly mean COD varied indistinctly during 2009–19 even though the linear trend has moved upward with increasing year as shown in Fig. 7a. The yearly mean COD increased from 15.9 to 30.8 mg/l during 2010–2012 and from 11.5 to 44.3 mg/l during 2013- 2015. Mean COD for the years 2018 and 2019 was 15.1 and 20.4 mg/l, respectively. The highest yearly mean COD was 44.33 mg/l for the year 2015 followed by the year 2012 with 30.8 mg/l, while the least mean COD value was observed in 2013 as 11.5 mg/l. A sharp decline in yearly mean COD was noted during the years 2012–13 and 2015–16. The monthly data of each month during 11 years period is shown in Fig. 7b. The width between the first and third quartiles for January, April, and May indicated maximum variation in COD values. The September quartile indicated less variation in COD as compared with the other months. The CODs for January and May had the highest median values of 27 and 24.5 mg/l, respectively, whereas April had the least median value of 8 mg/l. The median CODs for March, August, September, October, and November lay in the range of 11–16 mg/l. We found that the monthly mean CODs of May and November had maximum and minimum values of 33.9 and 14.2 mg/l, respectively.

The trend of COD followed a decreasing path moving from January to December. The monthly mean CODs of January, February, and May were found to be higher than 30 mg/l whereas for April, June, and October they lay between 20 and 30 mg/l, and for the remaining months mean COD was found to be lesser than 20 mg/l. A decline in the mean COD was observed from May to August, with a sharp increase in mean COD from March to May.

Figure 7c shows changing COD for each month as the year preceding. From 2009 to 2012, the COD values for January, February, March, and April were higher than 30 mg/l, whereas, since 2017, the COD values were seen well below 30 mg/l during these months. In May, an exceptional high COD of 125 mg/l was observed and for the same month, a continuous increase in COD was noted from the years 2013 to 2016. Also, the rise in COD was seen for June, August, October, and December during 2012–15. Excluding May, February, April, and September had maximum CODs of 85, 78, and 67 mg/l, respectively. During 2009–19, January and June to November showed a growing linear trend, whereas the rest of the months had a declining trend. Compared with the international standards, the annual and monthly mean CODs exceeded the WHO guideline and were classified as Classes III-V of UNECE standards. Also, the values were found well above the threshold value. During the years 2009, 2012, and 2013, the monsoon period showed COD value in Class II, while, during the overall monsoon period, water quality lay in Class III. During the year 2015, Yamuna, in terms of COD, was in the worst condition as it lied in Class V. Monthly variation COD for 2018–19 rarely plunged under WHO standards and represented to be in Classes III and IV.

### Total alkalinity

**Figure 8:**
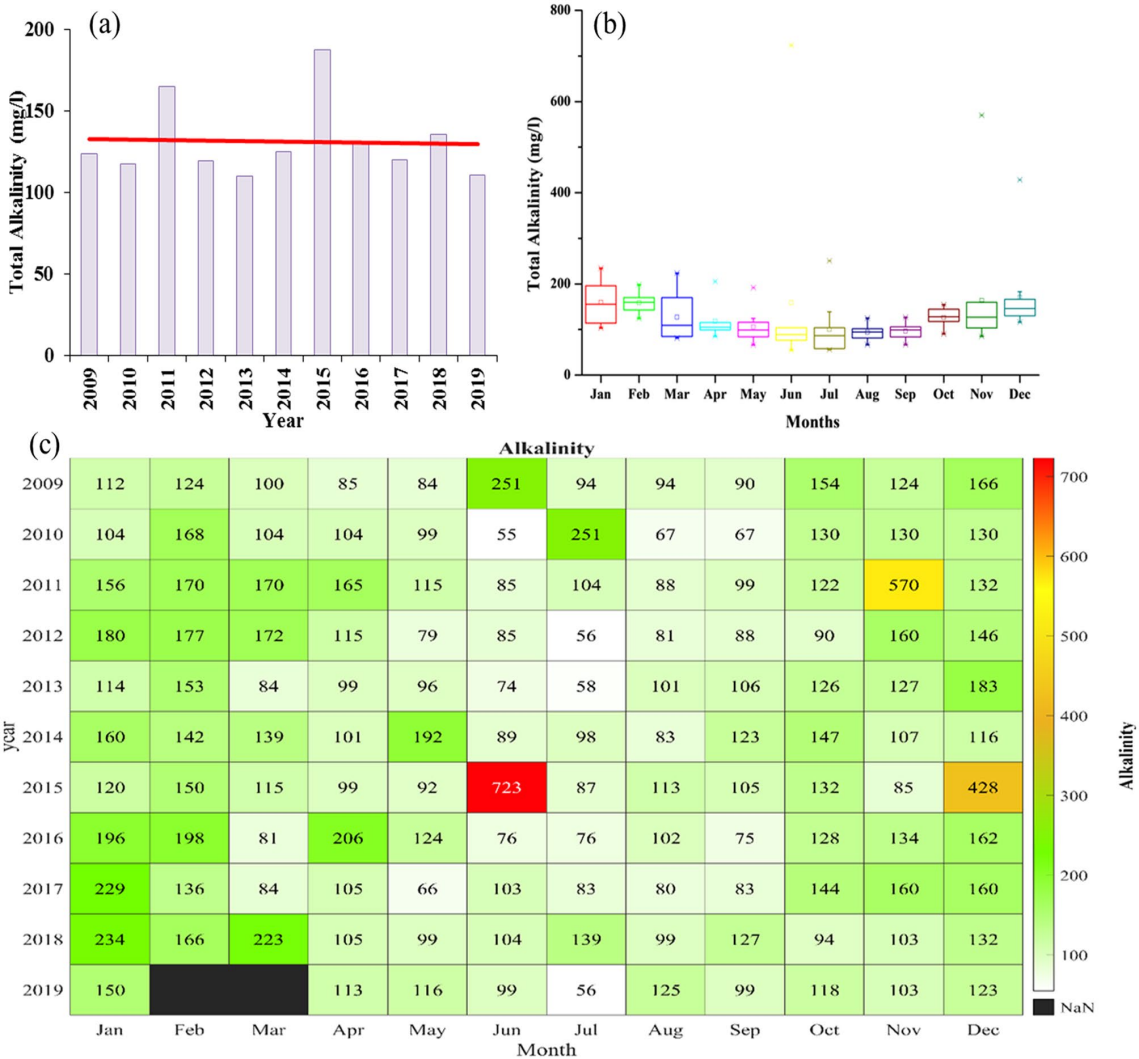
(**a**) Yearly variation of total alkalinity from 2009 to 2019. The red line shows a linear trend of total alkalinity. (**b**) Monthly variation of total alkalinity from 2009 to 2019. (**c**) Monthly mean variation of total alkalinity from 2009 to 2019.

Total alkalinity is mostly due to calcium carbonate (CaCO_3_) and also important for sustaining aquatic life. The yearly mean variation of total alkalinity during 2009–19 is shown in Fig. 8a. Total alkalinity was found to be the highest during 2015 followed by 2011 with values of 187.37 and 164.6 mg/l, respectively, while, for the rest of the year, the annual means were in the range of 110–150 mg/l. The linear trend for the yearly mean for the entire period remained constant. With these values of total alkalinity, the quality of river water lay in category II (UNECE 1994).

We have shown the monthly distribution of total alkalinity in Fig. 8b with the third quartile of each month lying below the mark of 200 mg/l. The median values dropped from January to July and then raised from July to December. The monthly mean total alkalinities in February and January showed the first and second-highest median values of 159.8 and 155.5 mg/l, respectively, while July and June showed the lowest median values of 86.5 and 88.9 mg/l, respectively. Median values during May, August, and September lay in the range of 90–100 mg/l, while for March, October, November, and December they were between 100 and 150 mg/l. The linear trend for monthly mean total alkalinity also followed the same pattern as the yearly mean. The total alkalinity declined from January to May from 159.4 to 105.5 mg/l and, then from August to December, it raised from 93.8 to 170.7 mg/l. August and December showed the lowest and highest values of total alkalinity, respectively.

The monthly variation in total alkalinity for each year during 2009–19 is shown in Fig. 8c. June (2015), November (2011), and December (2015) were the months with the highest total alkalinities of 723.3, 569.9, and 428.1 mg/l, respectively. During 2009–19, total alkalinities for February, March, May, July to September remained within 200 mg/l. The lowest total alkalinity of around 55 mg/l was noticed for June (2010) and July (2012). Although the linear trend for monthly total alkalinity showed almost the same slope except for January, July, and November. A wave pattern in total alkalinity for August, September, and October was noticed with an increasing trend during 2009–19. In 2019, the total alkalinity was below 150 mg/l throughout the year 2019. For maintaining WHO and BIS standards, the minimum total alkalinity must be 200 mg/l, while it was not possible to fulfill the standards for both monthly and yearly aspects in the study areas of concern. There were only a few months when total alkalinity was found to be higher than 200. Comparing with UNECE standard, 11 out of 12 monthly mean alkalinities lay in Class II and the remaining one month in Class III category. March 2018 was the last month since 2018 when Yamuna's total alkalinity was well above-mentioned the water standards.

### Total dissolved solids (TDS)

**Figure 9:**
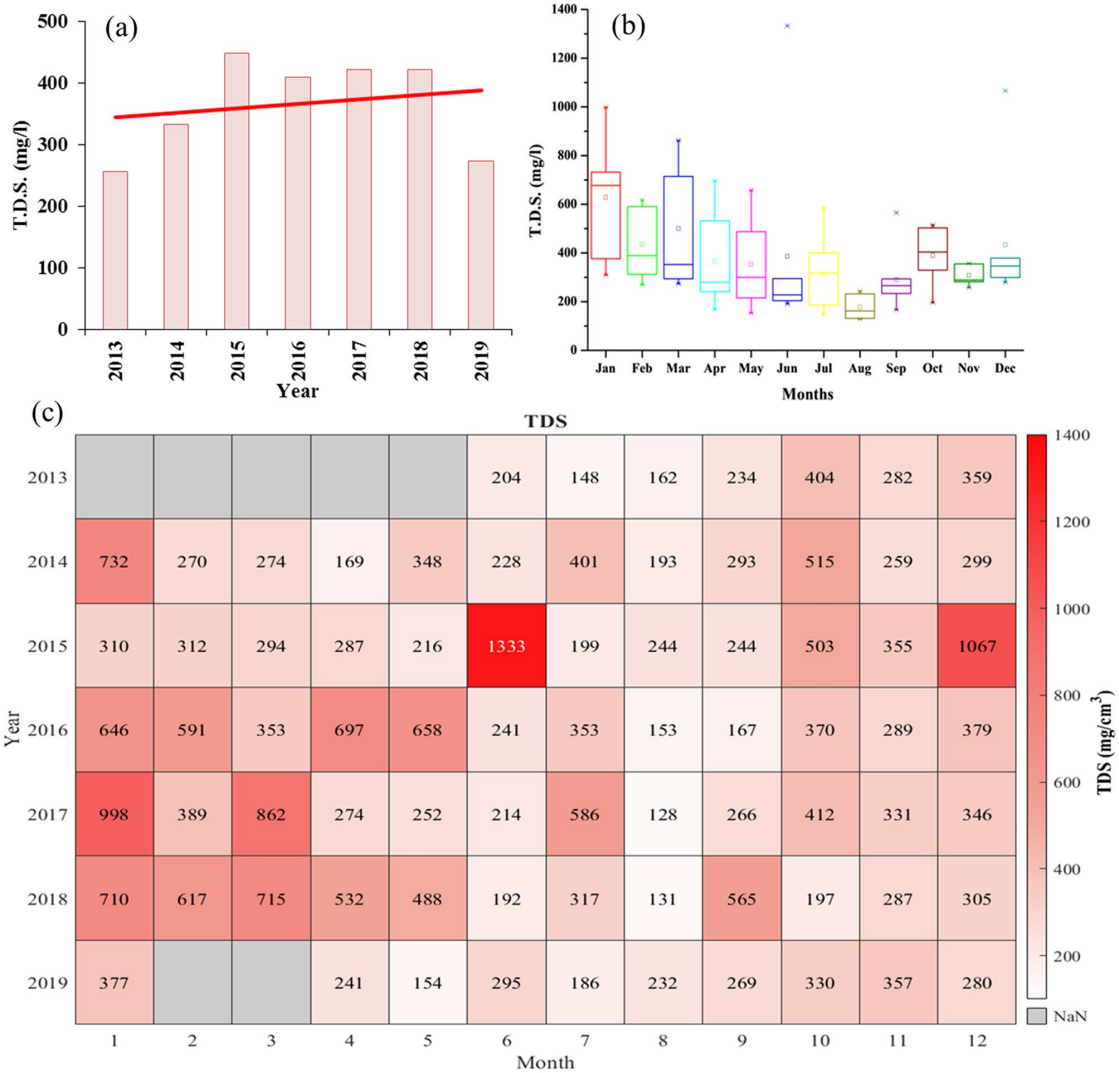
(**a**) Yearly variation of TDS from 2013 to 2019. The Red line shows a linear trend of TDS. (**b**) Monthly variation of TDS from 2013 to 2019. (**c**) Monthly mean variation of TDS from 2013 to 2019.

Total Dissolved Solids (TDS) define the presence of inorganic compounds along with organic matter in small concentrations originated by naturally, household, and industrial sources. The data was available from 2013 onwards. The yearly mean TDS was found to be highest in 2015 (447 mg/l) followed by 2017 and 2018 with 421 mg/l (Fig. 9a). The lowest yearly mean TDS was observed as 256 mg/l in 2010, while the recent value of 272 mg/l was observed in 2019. Although the linear trend for yearly TDS indicated the rise in overall TDS. A maximum drop in yearly TDS was observed with a fall of 36% during 2018–19.

The 25 to 75 percentiles of interquartile range of all twelve months for TDS are shown in Fig. 9b. Although March had the maximum width of interquartile range, the maximum median value of TDS was 678 mg/l for January. Except for August and October, the median TDS values for the rest of the months lay in the range of 200–400 mg/l. August had the lowest median TDS of 162 mg/l and October had 404 mg/l. A sharp decline in the trend of monthly mean TDS was observed during 2013–19, but mean TDS fluctuated throughout the year. Similarly, with median TDS, the monthly mean TDS for January and August had maximum-minimum mean values of 628.3 and 177.57 mg/l, respectively. Except for August, the monthly mean values of TDS were above the mark of 200 mg/l. From April to July, they lay in the range of 300–400 mg/l, while in February, March, and December, they ranged between 400 and 500 mg/l.

In February and March, TDS had a higher magnitude of rising, while it appeared to be almost constant in August as shown in Fig. 9c. In January, TDS was measured as 732 mg/l in 2014 and dropped to its lowest point of 310 mg/l in the next year, but then it reached 998 mg/l in 2017. The TDS values during June were well below the mark of 300 mg/l from 2009 to 2019, but during June 2015, the monthly mean TDS was found to be 1333 mg/l. This was the maximum value of TDS during the whole study period. For the same year, the second-highest TDS of 1067 mg/l was also observed in December. TDS remained mostly below 300 mg/l for August and September (mostly during monsoon months) with the lowest TDS of 128 mg/l in August 2017. In 2019, the TDS value mostly ranged between 200 and 300 mg/l. Yearly and monthly mean values of TDS were observed almost under WHO standards and in the Class A category of Indian standards. The TDS value was found below 500 mg/l during each monsoon season. During winter, summer, and post-monsoon months, the TDS of river water never exceeded the Class C category as per BIS standard and during January, the monthly mean average remained higher in comparison to other months.

### Total coliform

**Figure 10:**
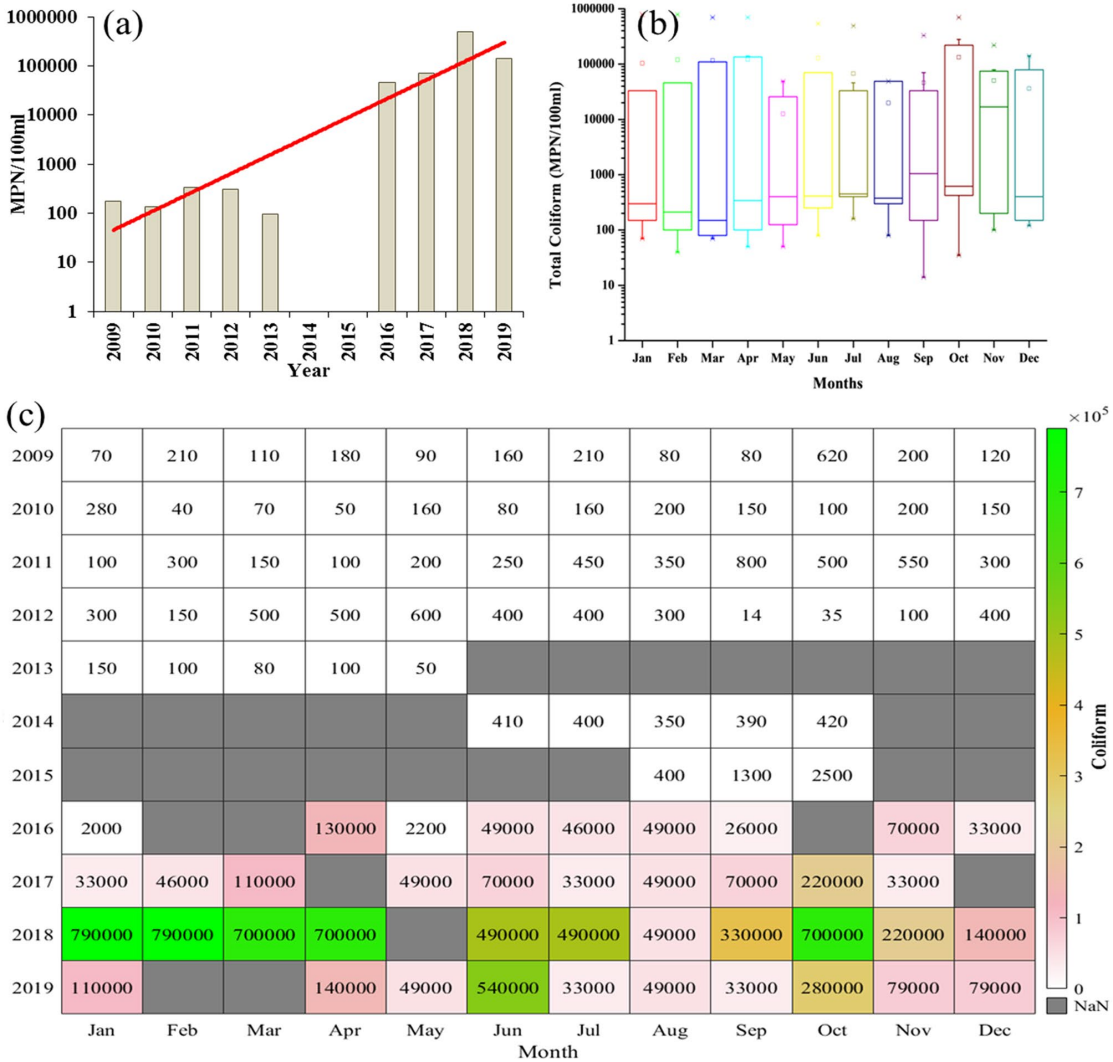
(**a**) Yearly variation of total coliform from 2009 to 2019. Redline shows an exponential trend of total coliform. (**b**) Monthly variation of total coliform from 2009 to 2019. (**c**) Monthly mean variation of total coliform from 2009 to 2019.

Human and animal discharges are the main source of fecal coliform bacteria whose excessive presence in water degrades the water quality. During 2009–19, there was an exponential rise in total coliform as shown in Fig. 10a. The yearly mean in 2009 was 177 MPN/100 ml, which in the decade reached 139,200 MPN/100 ml in 2019. The difference of yearly mean for two periods of 2009–13 and 2016–19 was more than 100 times the value at its starting period. The year 2018 was observed with the highest yearly mean of 490,818 MPN/100 ml, which was reduced in 2019, with the lowest total coliform count of 136.7 MPN/100 ml in 2010. Note that the years 2014–15 were excluded from comparison for this case due to less availability of data for the whole year.

Figure 10b shows a large monthly variation in total coliform in each month during 2009–19. The median of most of the months was below 500 MPN/100 ml, whereas median values were 1050, 620, and 16,775 MPN/100 ml for September, October, and November, respectively. The monthly mean for January, February, March, April, June, and October was above 100,000 MPN/100 ml. Maximum and minimum monthly means were observed for October (133,797 MPN/100 ml) and May (12,663 MPN/100 ml), respectively.

Figure 10c indicates the exponential rise in the trend of total coliform in each month since 2009. Total coliform counts during 2009–14 were well below the 500 MPN/100 ml mark, while some months also showed counts near 800 MPN/100 ml. However, from 2016 onwards, the counts crossed a sustainable mark of 5000 MPN/100 ml for every month of each year. From 2009 to 2013, the total coliform counts fell mostly in Class B but exceeded all limits of Indian water quality standards to a great extent during 2016–19. The monthly mean was not even close to the maximum total coliform limit of 5000 MPN/100 ml, which made water quality in Class D and E categories. WHO standards nullify the presence of fecal coliform in water, whereas the Yamuna River was found to be in its alarming situation for this particular parameter.

### Water discharge

**Figure 11:**
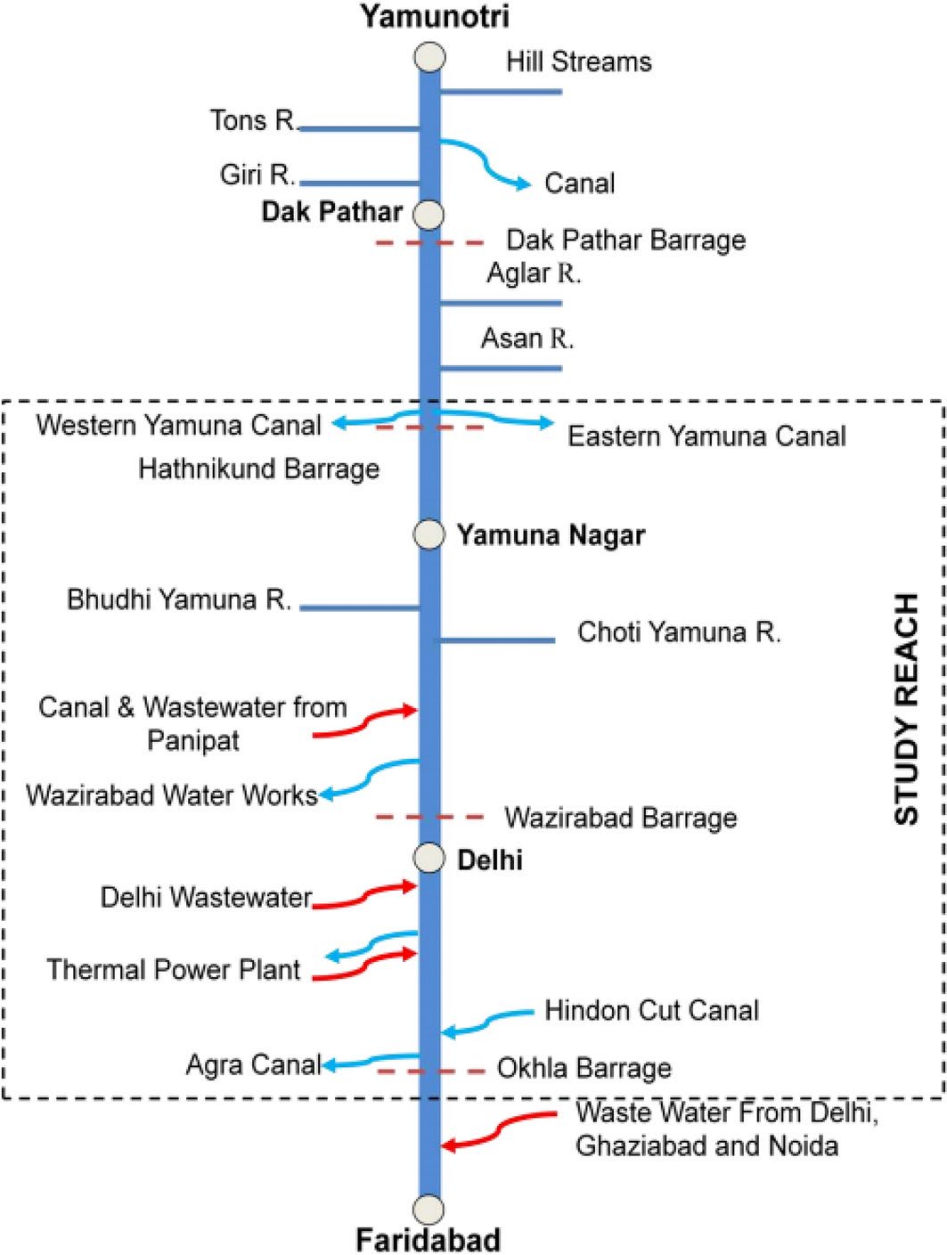
Points of water abstraction and additions in Yamuna river. (*Source*: https://yamuna-revival.nic.in/wp-content/uploads/2020/07/Final-Report-of-YMC-29.06.2020.pdf).

**Figure 12:**
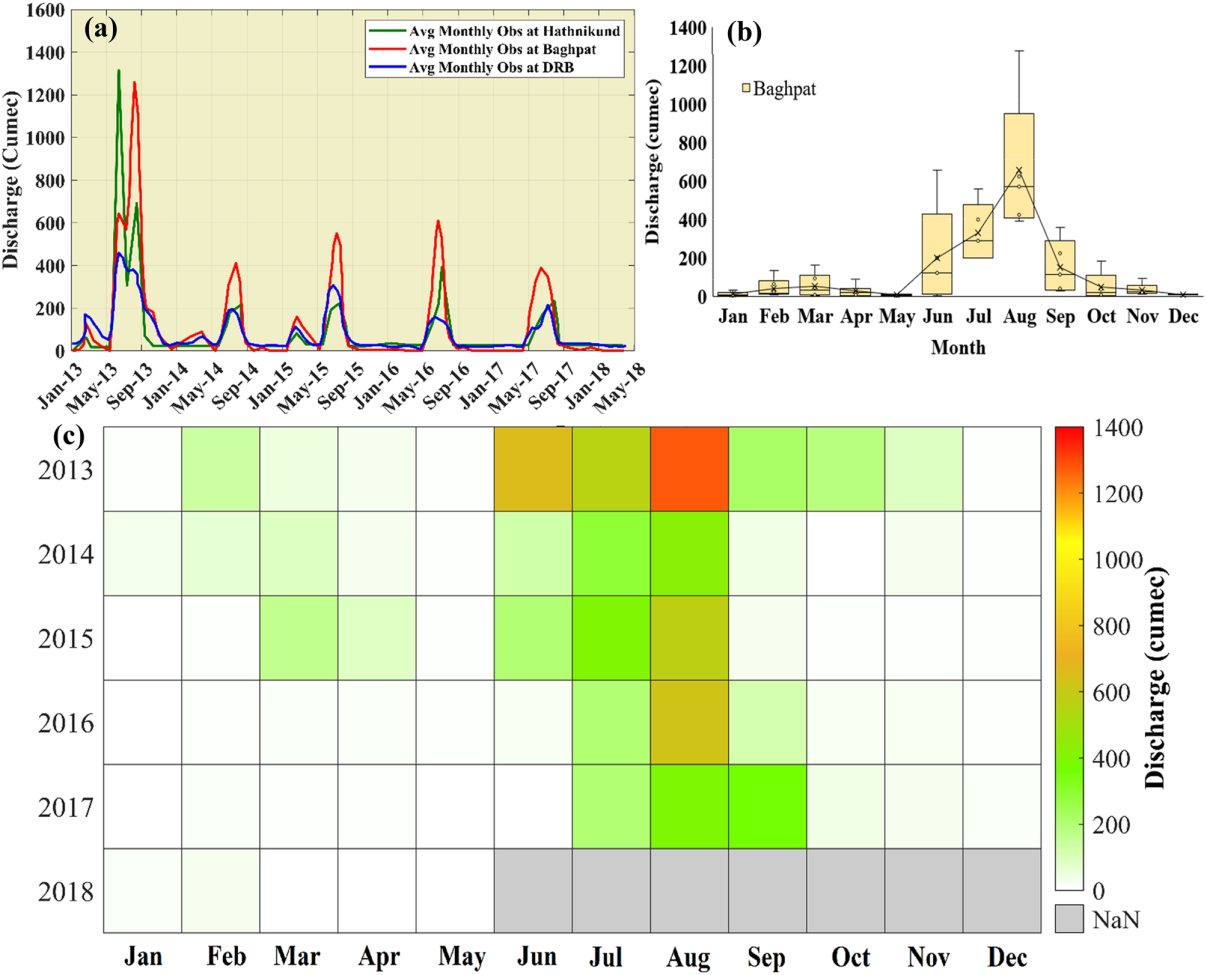
(**a**) Temporal variation of water discharge at Hathnikund, Baghpat, and Delhi Railway Bridge (DRB) stations during Jan 2013 to May 2018. (**b**) The distribution of monthly mean water discharge during Jan 2013 to May 2018 at Baghpat. (**c**) Monthly mean variation of discharge from 2013 to 2018.

The freshwater supply and inflow of wastewater in the river may affect the water quality. To uncover the influence, we analyzed the water discharge in the river Yamuna from 2013 to 2018 (the data is available for this period only). In Fig. 11, we show the major locations of water abstraction and confluence in the Yamuna river starting from Yamunotri to Faridabad stretch. Hathnikund barrage was constructed to regulate the Yamuna water supply to Haryana, Uttar Pradesh, and Delhi for agricultural and domestic purposes and it was also decided by the Government to maintain 10 cumes of water in the Yamuna downstream to maintain the aqua life in river. In Fig. 12, we show the water discharge data of Hathnikund, Baghpat, and DRB. From 2013 to 2018, a significant fall in the water discharge is observed especially at Hathnikund, which allows the upstream water to reach Palla. The mean discharge in the river was found to be 123.7 cumes during the whole study period and the minimum was found much lower than 10 cumes (as suggested by the Government of India). Also, a significant fall is observed in recent years. The mean water discharge values at Baghpat are found to be 225 cumes and sometimes they reached far below than 5 cumes.

**Figure 13:**
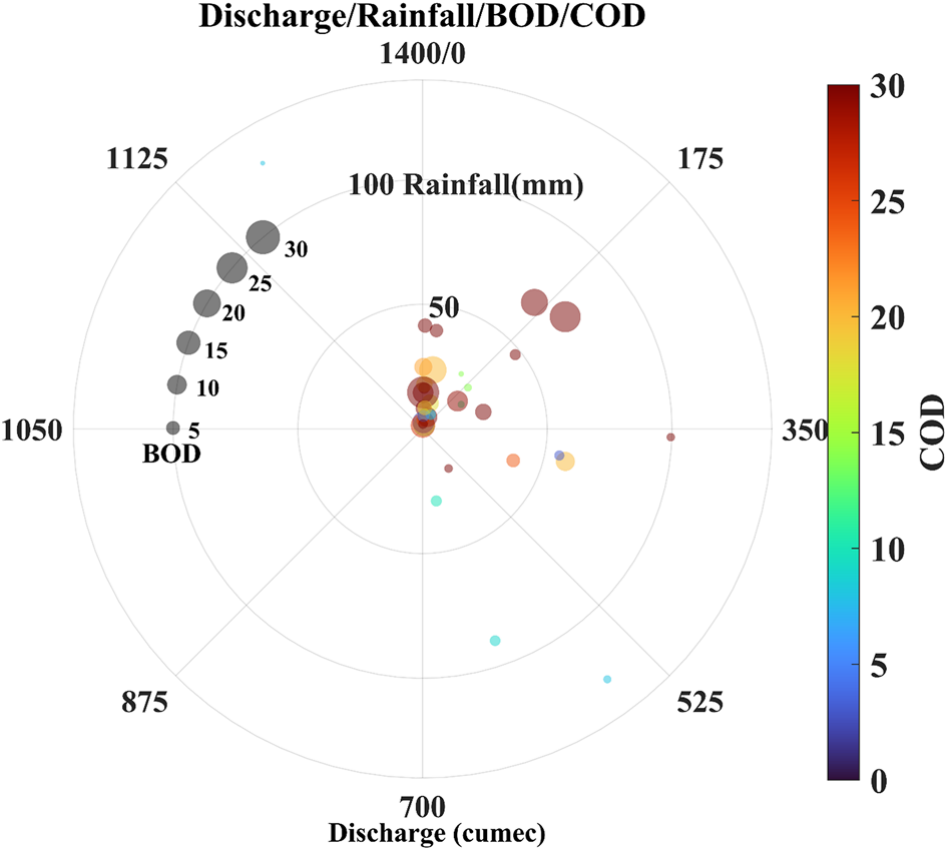
The relationship between the water discharge, Rainfall, BOD, and COD.

**Figure 14:**
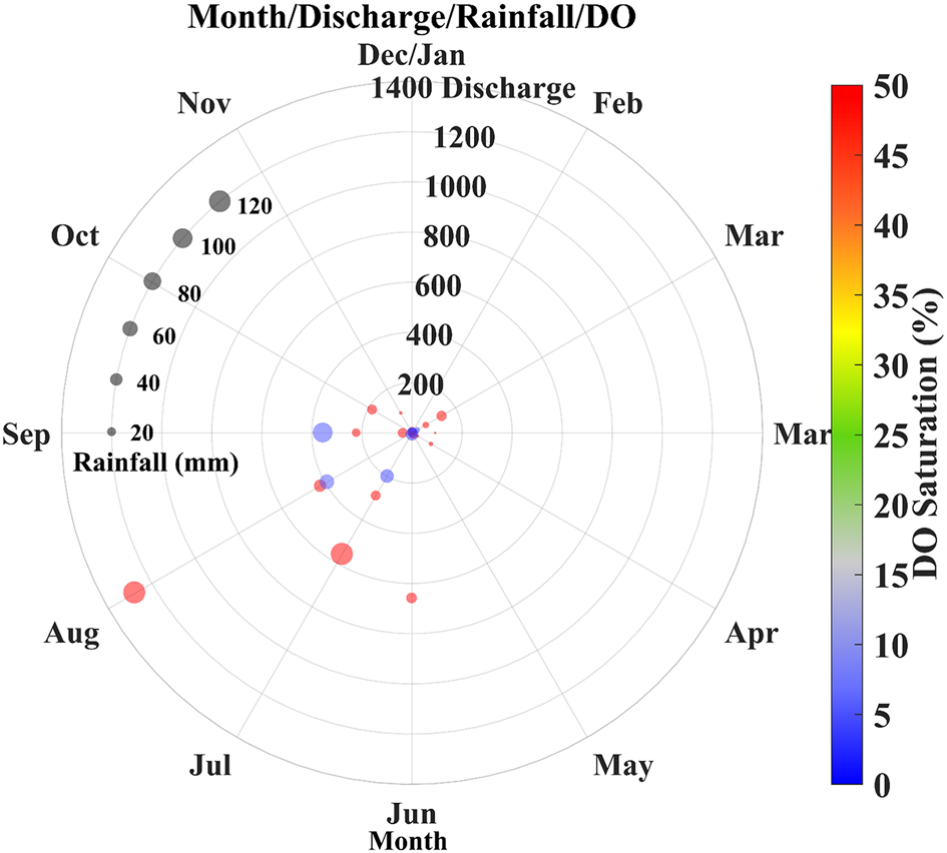
The monthly relationship between the water discharge, Rainfall and DO saturation.

At DRB, the mean discharge value is found to be 107 cumes. During monsoon months, the water discharge at Baghpat is higher than that at the Hathnikund so that the watershed of the Yamuna also helps in the rise of the water discharge due to rainfall and also major drains confluence in the Yamuna. We can see that during August, the water discharge is the highest followed by July and September. During January, May, and December, the water discharge values reached far below the prescribed limit, and hence sometimes during these seasons, the Yamuna almost dried up and only the sewage and the industrial wastes water flow during these months (Fig. 12c). After 2015, a significant fall in water discharge is observed during the summer and winter months. With the abstraction of fresh water at Hathnikund barrage and inflow of the drains, the water quality parameters are affected by a large extent in river Yamuna. Therefore, for further analysis of the impact of water discharge and rainfall, we also analyzed the relationship of monthly mean discharge, rainfall, DO saturation, BOD, and COD as shown by the polar plots (Figs. 13, 14).

### Variability of DO, BOD, and COD

Water quality can be affected by various factors. Hence, we investigated the variability of the DO, BOD, and COD with water discharge and rainfall. In Fig. 13, we plot the relationship of monthly mean water discharge at Baghpat, rainfall, BOD, and COD. The rainfall mostly affected the discharge values, but, sometimes, with low rainfall high discharge is observed. COD is mostly higher during the whole study period whereas BOD values have shown a fall in recent years. The impact of rainfall and high discharge is visible and low BOD and COD are observed. When the discharge is more than 525 cumes, the COD and BOD values are lower, and also more than 30 mm of rainfall is observed at that time. Further, we investigated the monthly relationship of the Discharge, rainfall, and DO saturation. Further analysis (Fig. 14) clearly shows that most rainfall occurred from June to September and caused a rise in the water discharge. During this time, the DO saturation values are more than 50%. The low DO saturation values are mostly observed during the time of low discharge and low rainfall (< 10%). We also found that during July to September with discharge between 200 to 400 cumes and rainfall more than 40 mm, the DO saturation remains lower than 10%. Further, Drain No. 6 having a catchment area of Samalkha Ganur and Sonipat, carries around 49 MLD of sewage in the year 2017 and rises to 210 MLD in the year 2018. Drain No. 8 crosses Drain No. 6 at Akbarpur, Sonipat, and meets river Yamuna just upstream of Palla village. There is a huge increase in the flow of Drain No. 8 between 2017 and 2018 from 196 to 2590 MLD. Drain No. 6 lined separately flow inside Drain No. 8 for 10 KM. Effluents from both drains mix with each other during the rainy season and due to accidental breach (https://sandrp.in/2015/04/13/blow-by-blow-how-pollution-kills-the-yamuna-river-a-field-trip-report/). Similarly, sewage flow of Drain No. 2, which meets river Yamuna 100 km upstream of Delhi is increased from 62 to 2092 MLD between 2017 and 2018 by Central Pollution Control Board of India. As per Haryana State Pollution Control Board (HSPCB), the capacities of Sewage treatment plants (STP) for Drains No. 2, 6, and 8 were 72, 104.5, 125.3 MLD, respectively, till 2018. Also, the capacities of the common effluent treatment plant (CETP) for Drains No. 2, 6, and 8 were 21, 33.2, and 10 MLD, respectively. Since the total capacity for treating wastewater was far beyond sewage generation during 2017–18 and hence the untreated water mixed with the river water. This rise in wastewater generation from industrial and urban areas has caused a drastic decrease in DO saturation and an increase in Total Coliform. As per CPCB 2018 report (https://yamuna-revival.nic.in/wp-content/uploads/2020/07/Final-Report-of-YMC-29.06.2020.pdf), 7-day average discharge of the 10-year return period (7Q10) does not meet the habitat requirements of the indicator fish species. These conditions show the impact of rapid urbanization and industrialization along the river bank with high carbon concentration. With the recent development in industrial regions, change in land use/land cover and rapid urbanization in the Haryana, the watershed of Yamuna suffered a lot and hence the water quality of the river. One of the major causes of the sudden fall in the DO saturation in recent years is the fall in the freshwater discharge at the Hathnikund and also the fall in the rainfall in recent years. Also, the deteriorating water quality of Yamuna is a major concern for the Government and mostly this is affected in the stretch between Hathnikund and Palla due to rise in inflow of untreated drains water supply. In recent years, the National Green Tribunal (NGT) of India also requested the states Government to take necessary actions to combat present situation of river Yamuna by installation of more STEPs, ETPs and maintaining the treatment capacity of present treatment plants and channelizing the sewage network to reach treatment plants properly. However, due to lack of adequate fresh water supply and mixing of untreated sewage through regulated and unregulated drains, the quality of the Yamuna river lies in critical conditions.

## Conclusion

Although National Capital Territory (NCT) is held responsible for most polluting river Yamuna, the study reveals that the quality of the river it receives is not admirable. The study of physiochemical and biological parameters shows variation in its monthly and yearly values during 2009–19. The effect of monsoon season can be easily seen on parameters like BOD, COD, total alkalinity, TDS and total coliform as their values declined, while DO saturation % showed a significant rise. DO saturation declined by more than 85% during this period. The BOD values improved during the last two years (2018–2019), but were still slightly higher than the permissible limit, while the COD value always remained quite higher than the permissible limits. In 2015, the worst condition was observed in terms of BOD and COD. Total alkalinity also remained low and below the prescribed standards, but TDS is the only parameter whose value was mostly in desired limits throughout the period. An exponential rise was observed in the total coliform count, which was 100–1000 times the maximum limit of IS:2296. Increasing discharge of partially treated industrial effluent and untreated sewage into the Yamuna in the past decade is considered to be the primary cause of the deterioration of water quality. Even after completion of YAP phases I and II, and ongoing phase III, the river still falls in the category of Class D or E under BIS specification, Class III or IV of UNECE standards, and does not fulfill the WHO guideline for water quality at Palla station.

## Data Availability

All the data used in the present study is freely available in the public domain and the web addresses are discussed in the manuscript, however, we will provide data to all the interested scientists.

## Abbreviations

BIS: Bureau of Indian Standards
BOD: Biological oxygen demand
CETPs: Common effluent treatment plants
COD: Chemical oxygen demand
CPCB: Central Pollution Control Board
CWE: Central Water Commission
DO: Dissolved oxygen
ETP: Effluent treatment plant
GEMS: Global Environmental Monitoring System
HSPCB: Haryana State Pollution Control Board
IMD: India Meteorological Department
ISRO: Indian Space Research Organization
KLD: Kiloliters per day
MEERA-2: Modern-Era Retrospective analysis for Research and Applications, Version 2
MINARS: Monitoring of Indian National Aquatic Resources
MLD: Millions of litres per day
MoEF: Ministry of Environment and Forests
MPN: Most probable number
NCRPB: National Capital Region Planning Board
NCT: National Capital Territory
NGT: National Green Tribunal
NWMP: National Water Quality Monitoring Programme
SPCB: State Pollution Control Board
TDS: Total dissolved solid
UNECE: United Nations Economic Commission for Europe
WHO: World Health Organization
WRIS: Water Resources and Information System
YAP: Yamuna Action Plan
